# Perineural Invasion in Vulvar Squamous-Cell Carcinoma Is an Independent Risk Factor for Cancer-Specific Survival, but Not for Locoregional Recurrence: Results from a Single Tertiary Referral Center

**DOI:** 10.3390/cancers14010124

**Published:** 2021-12-28

**Authors:** Leonardo Micheletti, Fulvio Borella, Mario Preti, Valentina Frau, Stefano Cosma, Sebastiana Privitera, Luca Bertero, Chiara Benedetto

**Affiliations:** 1 Division of Gynecology and Obstetrics 1, Department of Surgical Sciences, City of Health and Science University Hospital, University of Turin, 10126 Turin, Italy; leonardo.micheletti@unito.it (L.M.); mario.preti@unito.it (M.P.); dott.valentinafrau@gmail.com (V.F.); stefano.cosma@unito.it (S.C.); chiara.benedetto@unito.it (C.B.); 2 Pathology Unit, Department of Medical Science, University of Turin, 10126 Turin, Italy; sprivitera@cittadellasalute.to.it (S.P.); luca.bertero@unito.it (L.B.)

**Keywords:** vulvar squamous-cell carcinoma, vulvar cancer, perineural invasion, recurrence, survival, prognostic factors

## Abstract

**Simple Summary:**

Vulvar squamous cell carcinoma is a rare tumor but represents a serious health issue, especially due to the increasing incidence over the past decades. Many efforts have been made to identify new prognostic and therapeutic factors and, in this context, growing evidence concerning a pivotal role of perineural invasion. With this study, we investigated the role of perineural invasion in a large cohort of FIGO stage Ib-IIIc vulvar squamous cell carcinomas and found that perineural invasion-positive tumors have more aggressive biological behaviors and showed reduced cancer-specific survival as compared to perineural invasion-negative tumors, while this feature does not appear to be related to a greater risk to develop loco-regional recurrence. Further evaluations are warranted to confirm the prognostic role of perineural invasion and its potential use to tailor adjuvant treatment.

**Abstract:**

The aims of this study were to assess the prevalence of perineural invasion (PNI) in vulvar squamous cell carcinoma (VSCC) and its prognostic role in locoregional recurrence (LRR) and cancer-specific survival (CSS). We performed a retrospective analysis of 223 consecutive stage IB–IIIC surgically treated VSCCs at S. Anna Hospital, University of Turin, from 2000 to 2019. We identified 133/223 (59.6%) patients with PNI-positive VSCCs. PNI was associated with aggressive biological features (i.e., advanced FIGO stage, larger tumor diameter, greater depth of invasion, a higher number of metastatic lymph nodes, and lymphovascular invasion) and shorter 5-year CSS (78% vs. 90%, log-rank *p* = 0.02) compared with PNI-negative VSCCs. Multivariate analysis showed that PNI (HR 2.99 CI 95% 1.17–7.63; *p* = 0.02) and the presence of tumor cells on pathological surgical margins (HR 3.13 CI 95% 1.37–7.13; *p* = 0.007) are independent prognostic factors for CSS. PNI does not appear to be related to LRR, but is an independent prognostic factor for worse survival outcomes. Future studies are necessary to explore the possible value of PNI in tailoring the choice of adjuvant treatment.

## 1. Introduction

Vulvar cancer (VC), although rare, represents an increasingly serious threat to women’s health. The incidence rate of VC has progressively increased in recent decades in high-income countries—in particular among women aged < 60 years [1,2].

Vulvar squamous cell carcinoma (VSCC)—the most frequent histological type of VC—can be split into two main distinct entities according to its pathogenic mechanism: HPV-independent tumors, and HVP-related tumors [3]. The former represents the most common form, and is linked to chronic vulvar dermatosis such as lichen sclerosus; furthermore, this subtype of VSCC is often associated with differentiated vulvar intraepithelial neoplasia (dVIN) as a precursor alteration. The latter, on the other hand, is preceded by a well-defined premalignant lesion (vulvar high-grade squamous intraepithelial neoplasia—V-HSIL) that is linked to HPV infection in most cases [4].

More recently, VSCCs were divided according to the presence or absence of TP53 mutations with prognostic implications [5].

The prognosis of VSCC is mainly dependent on some tumor features, which are evaluated according to the 2014 International Federation of Gynecology and Obstetrics (FIGO) staging system [6], and encompass lesion dimension, depth of invasion (DOI), and metastasis of groin lymph nodes. Approximately 60% of VCs are diagnosed at an early stage (FIGO stage I/II), 28% with regional lymph nodes’ involvement, and 6% with distant metastasis [7]. 

Surgical excision represents the cornerstone treatment for VSCC and can range from a wide, deep, local excision to a total deep vulvectomy [8,9,10]. 

Adjuvant therapies such as chemotherapy and radiotherapy (RT) are effective options to prevent recurrence and improve survival outcomes for patients with VSCC; however, the recurrence and mortality rates remain high [11]. 

Several efforts have been made to find new prognostic factors to identify patients with an increased risk of recurrence or death who may benefit from adjuvant treatments [12,13,14,15,16]; in this context, the role of neoplastic perineural invasion (PNI) is emerging [17]. PNI is defined as the invasion of neoplastic cells through any of the three layers that constitute the nerve sheath (i.e., epineurium, perineurium, and endoneurium) [18]. The mechanisms favoring PNI are not fully understood, but they involve chemotactic and extracellular adhesion proteins, growth factors, and cells that compose the tumor microenvironment [18,19,20]. Growing evidence concerning SCC suggests a key role of PNI not only in shaping tumor progression but also in affecting survival [21,22,23,24]; however, few authors have investigated the prognostic role of PNI in VC [17].

The aim of this study is to evaluate the survival outcomes of a large series of consecutive VSCCs, focusing on the prognostic role of PNI.

## 2. Materials and Methods

We performed a retrospective study of all patients surgically treated for VSCC at the Department of Surgical Sciences, S. Anna Hospital, University of Turin, from 2000 to 2019.

The following clinical and pathological information was retrieved from medical charts: age at diagnosis, FIGO stage, tumor size, DOI (in mm), surgical margin status, tumor’s pattern (focal or multifocal), presence of nodal metastases, number of metastatic lymph nodes, extracapsular spread, bilateral groin involvement, associated lichen sclerosus, associated VIN (differentiated and V-HSIL), presence of PNI, presence of lymphovascular invasion (LVI), and adjuvant RT. 

Patients with VSCC FIGO stage IB/IIIC who underwent a radical total or partial vulvectomy or a wide local excision and bilateral inguinofemoral lymphadenectomy were considered for the analysis. We excluded the stage IA tumors (superficially invasive VSCC), since in a previous study by our group the presence of PNI was observed in a single case only [25]. Furthermore, patients with distant metastasis at the time of diagnosis were not considered.

The tumor size (in mm) was considered as a continuous and categorical variable analyzing the following cutoffs: 20 mm, 40 mm, and the mean value.

All hematoxylin- and eosin-stained slides were reviewed by a dedicated pathologist specialized in the field of lower genital tract diseases in order to confirm the diagnosis and the DOI, and to report the presence of LVI and/or PNI (if not reported in the original histological examination). In cases in which PNI status was not reported or was reported as absent in the original histological examination, double immunohistochemistry staining for S100 and cytokeratins (AE1/AE3) was performed. PNI-positive cases were defined according to the Liebig definition as the presence of tumor cells encompassing at least 33% of the nerve circumference or invading any of the three layers of the nerve sheath [26], while LVI was defined as the presence of cancer cells inside the capillary lumens of either the lymphatic or the microvascular drainage systems within the primary tumor. 

Regarding the surgical margins, different cutoffs have been proposed in order to define negative surgical margins [14,27,28,29,30]. According to the European Society of Gynecological Oncology Guidelines, we considered the surgical margin as negative if no tumor cells were detected on the margin at pathological examination [31]; however, we also analyzed the impact on survival outcomes of different cutoffs in terms of tumor cells’ distance from the margin (<3 mm, <5 mm, and <8 mm). 

All patients with >1 positive node and/or the presence of extracapsular invasion underwent adjuvant inguinal and pelvic RT.

Chemoradiotherapy with weekly cisplatin at 40 mg/m^2^ for 6 weeks was performed in patients with good performance status and a number of positive groin lymph nodes >2.

A follow-up visit was performed every 4 months during the first 3 years, then every 6 months for the subsequent 3 years, and then once every 12 months. An appointment for the next follow-up visit was made during each checkup. Additional clinical examinations were also carried out at the patient’s request. The follow-up visits were performed with vulvar and groin examination, along with biopsy in cases of any suspicious lesion(s), while in cases of suspicion of distant metastasis, further diagnostic tests were performed (abdominal/chest computed tomography and/or positron emission tomography, as appropriate). Tumor recurrence was defined as any histologically confirmed locoregional recurrence (LRR) of VC. 

We excluded patients without histological confirmation of VC or who had been surgically treated at other institutions, or with a follow-up shorter than 6 months. Disease status or cause of death was ascertained from clinical charts or cancer registry data of our region (Piedmont Cancer Registry, Centre for Epidemiology and Prevention in Oncology in Piedmont, Turin, Italy). All LRRs were treated surgically, whereas patients who developed distant recurrences received systemic treatment. All data were collected after pseudonymization in an institutional database including vulvar malignant neoplasms.

Ethical approval was not required due to the retrospective nature of the study, as stated by our institutional review board. Statistical analyses were performed using IBM^®^ SPSS^®^ v.25 (SPSS Inc., Chicago, IL, USA) software. Data were analyzed descriptively and represented as counts and percentages. Differences in proportions between patients who had an LRR and those who had not were tested using Pearson’s chi-squared test or Fisher’s exact test, as appropriate.

For continuous variables, the Shapiro–Wilk test was used to test the normality of distribution, while the Mann–Whitney U-test was used for comparison of data. Survival outcomes were analyzed by the Kaplan–Meier method and by univariate and Cox proportional hazards models and significant variables were included in the multivariate analysis. The analyses were conducted with a 95% confidence interval (CI), and a two-sided *p*-value of 0.05 was considered statistically significant.

## 3. Results

### 3.1. Clinicopathological Features

We included 223 consecutive stage IB–IIIC VSCCs surgically treated at our institution. All demographic, clinical, and pathological features of the entire cohort are summarized in Table 1 according to the presence or absence of PNI. The presence of PNI was initially reported in 96 cases of VSCC (43%); in the remaining cases, double immunohistochemistry staining for S100 and cytokeratins (AE1/AE3) enabled the identification of 37 additional cases of PNI-positive tumors (17%).

The mean age at diagnosis was 69.5 years (standard deviation ±11, range 27–89).

Approximately half of the patients in this cohort had FIGO stage III tumors (52%). The mean tumor size was 29 mm (standard deviation ± 18, range 1–130), whereas patients who had a tumor larger than 20 mm, 29 mm, or 40 mm accounted for 65% (145/223), 47% (105/223), and 27% (60/223) of patients, respectively. The mean DOI was 8 mm (standard deviation ± 6.5, range 2–55), while cases with DOI > 8 mm accounted for 41% of patients (92/223). Patients who had a histopathological tumor-free minimum margin distance <8 mm, <5 mm, <3 mm, and with tumor on the surgical margin accounted for 65% (131/201), 46% (93/201), 30% (60/201), and 15% (31/201) of patients, respectively.

Most patients had a unifocal lesion (90%, 220/223) and a number of metastatic groin lymph nodes ≤ 1 (75%, 167/223). In the case of metastatic groin lymph nodes, extracapsular cancer spread was observed in 29/109 patients (27%), while the number of patients with bilateral groin involvement was 35/109 (32%). Associated lichen sclerosus was found in 126/223 (56%) cases, while VIN was found in 58/223 (26%) cases. PNI and LVI were observed in 133/223 (59.6%) and 36/223 (16%) cases, respectively. Adjuvant RT was performed in 84/223 (37.6%) patients, while cisplatin in addition to RT was administered in 17/223 (7.6%).

Considering PNI as an independent variable, we observed that tumors with PNI had a more advanced FIGO stage (III vs. Ib-II) (*p* = 0.004), larger diameter, greater depth of invasion, a higher number of positive lymph nodes (*p* = < 0.001), and a greater proportion of LVI (*p* = 0.02) (Table 1).

No significant differences in distribution for any of the considered variables were observed between patients who had LRR and patients who did not; however, patients who died from VC-related causes compared with patients without evidence of disease or who died from other causes showed a higher prevalence of FIGO stage III tumors (*p* = 0.02), tumor involvement on the surgical margins (*p* = 0.01), metastatic lymph nodes (*p* = 0.01), and positive PNI (*p* = 0.002) (Table 2).

### 3.2. Survival Outcomes

Regarding survival results, the mean follow-up time was 49 months (median 31, standard deviation ± 48, range 6–224). Of the 223 patients analyzed, 90 (40%) developed local vulvar recurrences and 51 (23%) died from VC-related causes.

By univariate analysis, only advanced FIGO stage was related to LRR (hazard ratio (HR): 1.58, CI 1.04–2.46, *p =* 0.03); therefore, a multivariate analysis was not performed. Instead, considering the CSS, the following variables were related to a greater risk of VC-related death: FIGO stage III (HR: 2.55, CI 1.45–4.50, *p* = 0.001), higher DOI (HR: 1.05, CI 1.01–1.10, *p* = 0.009), DOI ≥ 8 mm (HR: 1.82, CI 1.05–3.16, *p* = 0.03), surgical margin involvement (HR: 1.88, CI 1.10–3.55, *p* = 0.04), number of positive groin lymph nodes > 1 (HR: 2.84, CI 1.59–5.06, *p* = < 0.001), bilateral groin involvement (HR: 2.06, CI 1.02–4.16, *p =* 0.04), and presence of PNI (HR: 2.75, CI 1.41–5.36, *p* = 0.003) (Table 3). According to multivariate analysis, surgical margin involvement and PNI were independent unfavorable prognostic parameters for CSS (HR: 3.13, CI 1.37–7.13, *p* = 0.007 and HR: 2.99, CI 1.17–7.63, *p* = 0.02, respectively). Conversely, FIGO stage, DOI, the number of positive groin lymph nodes, and bilateral groin involvement were not significant (Table 4).

**Figure 1:**
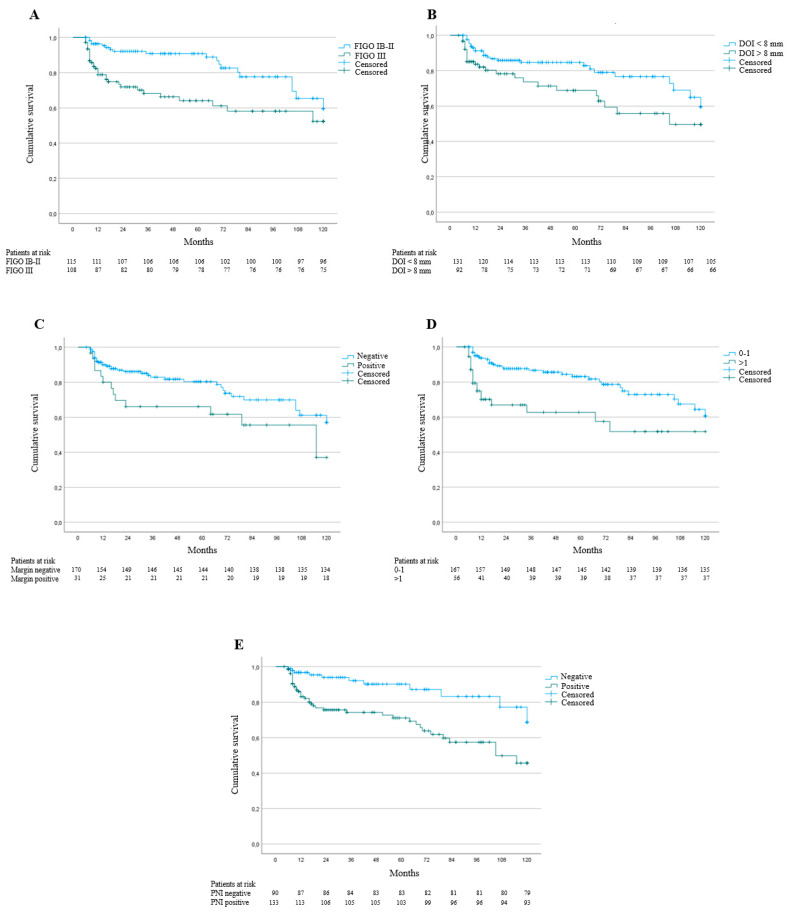
Kaplan–Meier curves for cancer-specific survival (CSS) according to FIGO stage (**A**), depth of invasion (DOI) (**B**), surgical margin involvement (**C**), number of positive groin lymph nodes (**D**), and perineural invasion (PNI) (**E**).

Finally, the following variables were related to 5-year CSS (Figure 1): FIGO stage, IB-II vs. III (5-year CSS: 92% vs. 73%, log-rank *p* = < 0.001); DOI, < 8 mm vs. > 8 mm (5-year CSS: 86% vs. 78%, log-rank *p* = 0.02); surgical margin involvement, yes vs. no (5-year CSS: 85% vs. 68%, log-rank *p* = 0.049); positive groin lymph nodes, 0–1 vs. > 1 (5-year CSS: 87% vs. 70%, log-rank *p* = < 0.001); PNI status, negative vs. positive (5-year CSS: 90% vs. 78%, log-rank *p* = 0.02).

## 4. Discussion

To date, there are no agreed protocols for the adjuvant treatment of VC—especially regarding the role of systemic chemotherapy; in fact, the choice of treatment is based on the assessment of pathological features such as DOI, surgical margin status, and nodal involvement [32].

In this context, multiple systemic chemotherapy regimens have been proposed, with the aim of making tumor cells more sensitive to RT; cisplatin-based chemotherapeutic regimens are the most frequently used [31,33,34]. 

Several efforts have been made to identify new prognostic factors to facilitate the choice of adjuvant therapy, but conflicting results have been reported [14].

In this context, PNI represents a key pathological feature of many solid malignancies, and is associated with poor survival outcomes in head and neck, pancreatic, prostate, colorectal, esophageal, and gastric cancers [35]. Regarding head and neck SCC, some authors found that the presence of PNI was related to an increase in LRR and reductions in disease-free interval (DFS) and overall survival (OS) [18,36]. 

A correlation between PNI, reduced survival, and increased risk of recurrence was also observed for pancreatic [37], gastric [38], and prostate cancers [39].

An emerging relationship between PNI and survival outcomes has also been proposed in recent years for some gynecological tumors, such as cervical cancer and VC.

For cervical cancer, a systematic review and meta-analysis showed that tumors with PNI had a significantly lower OS rate, while the presence of PNI was not related to DFS [40].

Regarding VSCC, the prevalence of PNI reported in the literature is variable, ranging from 8.7% to 52.4%; in our study, we observed a slightly higher presence of PNI (59.6%) than that reported by Holtoff et al. (52.4%) [41]. Wide variations in PNI rates have also been reported in other tumor types—for example, head and neck cancer, in which the reported PNI prevalence ranges from 5.2% to 90% [18].

Manifold reasons could explain the variations in PNI rates observed between studies. PNI evaluation and reporting are not always considered during the routine histopathological assessment of VSCC, and no international consensus has yet been reached. To date, only the Gynecological Oncology Working Group (AGO) of the German Cancer Society (DKG) and the German Society for Gynecology and Obstetrics (DGGG) have suggested determining PNI, even though its presence does not alter adjuvant treatment choice [42].

Regarding PNI assessment, our study defined PNI as described by Liebig et al. [26]; however, it must be noted that the required cutoff value of 33% nerve involvement by the tumor is a complex and empirical determination and, as expected, interobserver variability has been reported even among board-certified pathologists [43,44].

Additional immunohistochemical staining can better highlight PNI; studies that reported a higher prevalence of PNI in VSCC had used immunohistochemical analysis in addition to hematoxylin and eosin alone [41,45,46]. For example, in a study on oral cavity SCC, the revaluation of a case series using anti-S100 immunohistochemistry increased PNI detection from 30% to 82% [47].

Similarly to other authors, we found that PNI was associated with more advanced stages [46,48,49], larger tumors [45,49], greater DOI [41,45], greater lymph node involvement [45,48,50], and LVI [45,46,48,49]; these features suggest that the presence of PNI is associated with more aggressive biological behavior.

Studies on the survival impact of PNI in VC have analyzed DFS, including both local and systemic recurrences, and some authors have found that PNI is an independent prognostic factor for DFS [41,46,48].

PNI was not found to be significantly associated with DFS in terms of LRR. The fact that PNI was not linked to an increased risk of LRR can be explained in several ways. In an animal model of pancreatic cancer, it has been observed that neoplastic cells invading nerve structures can reach the extrapancreatic neural plexi and other distant sites through the perineural space [51]. In another animal model, it was observed that within 6 weeks of resection of the primary pancreatic tumor, 80% of cases developed retroperitoneal metastases [52]. The presence of metastases in nerve plexi distant from the primary tumor site has also been observed in clinical settings [26].

Our study is the first to evaluate the association between CSS and PNI instead of OS. CSS was analyzed because we considered it a more accurate outcome for evaluating a potential prognostic variable, since it removes competing causes of death [53,54]. Our data showed that PNI is strongly related to CSS. Other authors have also reported an association between PNI positivity and OS according to multivariate [46,48] or univariate analysis [45,49,55].

These results suggest that the nerves may represent a reservoir of neoplastic cells that can migrate to invade distant sites, and could explain how in our series PNI was not a risk factor for LRR, but only for CSS.

A summary of the most relevant studies regarding the role of PNI in VSCC is provided in Table 5.

The strengths of this study include a large cohort of consecutive patients with long-term follow-up, the review of histologic slides performed by an expert pathologist using immunohistochemical staining to increase sensitivity, and the use of CSS as the main survival outcome. Furthermore, all cases were surgically treated by the same surgeon with a systematic and standardized groin staging (total bilateral lymphadenectomy) [56].

The main limitation of this study is related to the potential differences in terms of patients’ management over the years, which was tailored accordingly to their clinical features.

## 5. Conclusions

We report how the presence of PNI in VSSCs is not related to LRR, but is associated with aggressive tumor features and poorer CSS, thus representing an unfavorable prognostic factor.

Unfortunately, all previous data reporting worse survival outcomes among cases harboring PNI were obtained from retrospective and monocentric studies. Prospective and multicenter studies by referral centers for the treatment of VC would enable its inclusion in therapeutic algorithms, although there are no ongoing clinical trials focused on PNI to date. Moreover, standardization of histological PNI assessment is also needed.

Furthermore, it is possible that the tumor-invaded nerve can serve as a reservoir for neoplastic cells and modulate the immune response by protecting cancer cells and favoring the aggressiveness of the tumor. Intriguing preclinical studies have identified potential therapeutic targets; for example, the secretion of glial-cell-derived neurotrophic factor (GDNF) in murine models has been correlated with a greater presence of neoplastic cells along nerves [57]. In another preclinical study, a role of Schwann cells in nerve invasion was suggested, as it was observed that these cells can migrate toward pancreatic and colon cancer cells. Interestingly, this process can be inhibited by blocking p75 (NTR) signaling in both Schwann and pancreatic neoplastic cells [58].

Additionally, with the rapid development of precision medicine for VC [59,60,61], basic and translational research is needed in order to better understand the mechanisms underlying PNI, so as to identify possible therapeutic targets [36].

## Figures and Tables

**Table 1:** Demographic, clinical, surgical, and histopathological characteristics of the whole cohort, and according to the presence of PNI. (Statistically significant factors are highlighted in bold).

Variable		All Patients N = 223	PNI-Negative N = 90	PNI-Positive N = 133	*p*-Value
**Mean age at diagnosis (range)**		69 ± 11 (27–89)	70 ± 13 (27–89)	69 ± 12 (39–86)	0.68
**Age ≤ 70 years**	Yes	106 (47%)	39 (37%)	67 (63%)	0.302
	No	117 (53%)	51 (44%)	66 (56%)	
**FIGO stage**	I–II	115 (52%)	57 (50%)	58 (50%)	**0.004**
	III	108 (48%)	33 (31%)	75 (69%)	
**Mean tumor size (mm) (range)**		29 ± 18 (1–130)	22 ± 14 (1–80)	32 ± 19 (5–130)	**<0.001**
**Tumor size ≤ 29 mm** **(mean value)**	Yes	118 (53%)	61 (52%)	57 (48%)	**<0.001**
	No	105 (47%)	29 (28%)	76 (72%)	
**Tumor size < 20 mm**	Yes	78 (35%)	44 (56%)	34 (44%)	**0.001**
	No	145 (65%)	46 (32%)	99 (68%)	
**Tumor size < 40 mm**	Yes	163 (73%)	78 (48%)	85 (52%)	**<0.001**
	No	60 (27%)	12 (20%)	48 (80%)	
**DOI (mean)**		8 ± 6.5 (2–55)	6 ± 5 (2–23)	9 ± 7 (2–55)	**0.001**
**DOI < 8 mm** **(mean value)**	Yes	131 (59%)	65 (50%)	66 (50%)	**0.001**
	No	92 (41%)	25 (27%)	67 (73%)	
**Surgical margin (tumor on margin)** **(missing 22)**	Yes	31 (25%)	12 (39%)	19 (61%)	0.94
	No	170 (85%)	37 (39%)	103 (61%)	
**Surgical margin < 3 mm** **(missing 22)**	Yes	60 (30%)	24 (40%)	36 (60%)	0.89
	No	141 (70%)	55 (39%)	86 (61%)	
**Surgical margin < 5 mm** **(missing 22)**	Yes	93 (46%)	34 (37%)	59 (63%)	0.46
	No	108 (54%)	45 (42%)	63 (58%)	
**Unifocal lesion**	Yes	200 (90%)	83 (41%)	117 (59%)	0.30
	No	23 (10%)	7 (30%)	16 (70%)	
**Positive lymph nodes**	0–1	167 (75%)	78 (47%)	89 (53%)	**0.001**
	>1	56 (25%)	12 (21%)	44 (79%)	
**Extracapsular spread**	Yes	29 (27%)	5 (17%)	24 (83%)	0.06
	No	80 (73%)	29 (36%)	51 (64%)	
**Bilateral groin involvement**	Yes	35 (32%)	9 (26%)	26 (74%)	0.39
	No	74 (68%)	25 (34%)	49 (66%)	
**Associated lichen sclerosus**	Yes	126 (57%)	46 (36%)	80 (64%)	0.18
	No	97 (43%)	44 (45%)	53 (55%)	
**Associated VIN**	No	165 (75%)	60 (35%)	105 (64%)	0.09
	V-HSIL	28 (12%)	13 (46%)	15 (54%)	
	Differentiated	30 (13%)	17 (57%)	13 (43%)	
**LVI**	Yes	38 (17%)	9 (23%)	29 (76%)	**0.02**
	No	185 (83%)	81 (44%)	104 (56%)	
**Radiotherapy**	Yes	84 (38%)	28 (33%)	56 (67%)	0.09
	No	139 (62%)	62 (45%)	77 (55%)	
**Cisplatin**	Yes	17 (8%)	9 (53%)	8 (47%)	0.27
	No	206 (92%)	81 (39%)	125 (61%)	

DOI: depth of invasion; FIGO: International Federation of Gynecology and Obstetrics; LVI: lymphovascular invasion; PNI: perineural invasion; VIN: vulvar intraepithelial neoplasia; V-HSIL: vulvar high-grade squamous intraepithelial neoplasia.

**Table 2:** Demographic, clinical, surgical, and histopathological characteristics according to locoregional recurrence (LRR) and cancer-specific survival (*n* = 223). (Statistically significant factors are highlighted in bold).

Variable		No LRR N = 133	LRR N = 90	*p*-Value	Alive, or Dead from Other Cause N = 172	Dead from Disease N = 51	*p*-Value
**Mean age at diagnosis (standard deviation, range)**		69.5 ± 12.7 (27–89)	69.2 ± 9.6 (43–86)	0.88	69.5 ± 12.0 (27–89)	61.5 ± 9.7 (43–86)	0.86
**Age ≤ 70 years**	Yes	62 (58%)	44 (42%)	0.74	91 (78%)	26 (22%)	0.92
	No	71 (61%)	46 (39%)		81 (76%)	25 (26%)	
**FIGO stage**	I-II	70 (61%)	44 (39%)	0.51	95 (83%)	19 (17%)	**0.02**
	III	63 (58%)	46 (42%)		77 (71%)	32 (29%)	
**Mean tumor size (mm) (standard deviation, range)**		30.4 ± 18.9 (1–130)	25.9 ± 15.9 (1–80)	0.07	28.2 ± 18.2 (1–130)	28.2 ± 16.7 (7–80)	0.54
**Tumor size ≤ 29 mm (mean value)**	Yes	64 (54%)	54 (46%)	0.08	94 (79%)	24 (21%)	0.34
	No	69 (66%)	36 (34%)		78 (73%)	27 (27%)	
**Tumor size < 20 mm**	Yes	42 (54%)	36 (46%)	0.19	63 (81%)	15 (19%)	0.34
	No	91 (63%)	54 (37%)		109 (75%)	36 (25%)	
**Tumor size < 40 mm**	Yes	92 (56%)	71 (44%)	0.10	126 (77%)	37 (23%)	0.92
	No	41 (68%)	19 (32%)		46 (77%)	14 (23%)	
**Mean DOI (mm) (standard deviation, range)**		8.4 ± 6.2 (2–40)	7.3 ± 6.9 (2–55)	0.23	7.8 ± 5.9 (2–40)	8.8 ± 8.2 (2–55)	0.34
**Depth of invasion < 8 mm** **(mean value)**	Yes	73 (56%)	58 (44%)	0.15	105 (80%)	26 (20%)	0.20
	No	60 (65%)	32 (35%)		67 (73%)	25 (27%)	
**Surgical margin (tumor on margin) (missing 22)**	Yes	14 (45%)	17 (55%)	0.10	18 (58%)	13 (42%)	**0.01**
	No	103 (61%)	67 (39%)		134 (79%)	36 (21%)	
**Surgical margin < 3 mm** **(missing 22)**	Yes	34 (57%)	26 (43%)	0.77	43 (72%)	17 (28%)	0.39
	No	83 (59%)	58 (41%)		109 (77%)	32 (23%)	
**Surgical margin < 5 mm** **(missing 22)**	Yes	53 (57%)	40 (43%)	0.74	68 (73%)	25 (27%)	0.44
	No	64 (59%)	44 (41%)		84 (78%)	24 (22%)	
**Unifocal lesion**	Yes	118 (59%)	82 (41%)	0.56	153 (76%)	47 (24%)	0.51
	No	15 (65%)	8 (35%)		19 (83%)	4 (17%)	
**Positive lymph nodes**	0–1	100 (60%)	67 (40%)	0.90	135 (81%)	32 (19%)	**0.02**
	>1	33 (59%)	23 (41%)		37 (66%)	19 (34%)	
**Extracapsular spread**	Yes	20 (69%)	9 (31%)	0.15	22 (76%)	7 (24%)	0.47
	No	43 (54%)	37 (46%)		55 (69%)	25 (31%)	
**Bilateral groin involvement**	Yes	20 (57%)	15 (43%)	0.92	21 (60%)	14 (40%)	0.13
	No	43 (58%)	31 (42%)		56 (76%)	18 (24%)	
**Associated lichen sclerosus**	Yes	71 (56%)	55 (44%)		74 (76%)	23 (24%)	0.79
	No	62 (64%)	35 (36%)		98 (78%)	28 (22%)	
**Associated VIN**	No	98 (59%)	67 (41%)	0.52	125 (76%)	40 (24%)	0.51
	V-HSIL	19 (68%)	9 (32%)		24 (86%)	4 (14%)	
	Differentiated	16 (53%)	14 (47%)		23 (77%)	7 (23%)	
**LVI**	Yes	23 (64%)	13 (36%)	0.60	28 (74%)	10 (26%)	0.48
	No	106 (59%)	73 (41%)		144 (78%)	41 (22%)	
**PNI**	Yes	75 (56%)	58 (44%)	0.22	93 (70%)	40 (30%)	**0.002**
	No	58 (62%)	32 (36%)		79 (88%)	11 (12%)	
**Radiotherapy**	Yes	49 (58%)	35 (42%)	0.75	64 (76%)	20 (24%)	0.79
	No	84 (60%)	55 (40%)		108 (77%)	31 (23%)	
**Cisplatin**	Yes	12 (71%)	5 (29%)		15 (88%)	2 (12%)	
	No	121 (59%)	85 (41%)		157 (76%)	49 (24%)	

DOI: depth of invasion; FIGO: International Federation of Gynecology and Obstetrics; LVI: lymphovascular invasion; PNI: perineural invasion; VIN: vulvar intraepithelial neoplasia; V-HSIL: vulvar high-grade squamous intraepithelial neoplasia.

**Table 3:** Univariate analysis of factors associated with locoregional recurrence (LRR) and cancer-specific survival (CSS) (statistically significant factors are highlighted in bold).

Variable			LRR			CSS	
		HR	95% CI	*p*-Value	HR	95% CI	*p*-Value
**Age**		1.006	0.98—1.02	0.50	1.008	0.98–1.03	0.56
**Age > 70**		1.10	0.73–1.67	0.63	1.13	0.65–1.94	0.67
**FIGO Stage III**		**1.58**	**1.04–2.46**	**0.03**	**2.55**	**1.45–4.50**	**0.001**
**Mean tumor size**		0.99	0.98–1.005	0.20	1.007	0.99–1.02	0.34
**Tumor size > 30 mm**		1.28	0.84–1.96	0.24	1.37	0.79–2.37	0.25
**Tumor size ≥ 20 mm**		1.09	0.72–1.67	0.66	1.55	0.85–2.83	0.15
**Tumor size ≥ 40 mm**		1.3	0.83–2.30	0.20	1.16	0.63–2.11	0.62
**DOI**		1.004	0.96–1.05	0.83	**1.05**	**1.01–1.10**	**0.009**
**DOI ≥ 8 mm**		0.94	0.61–1.45	0.80	**1.82**	**1.05–3.16**	**0.03**
**Surgical margin** **(tumor on margin)**		1.28	0.80–2.05	0.28	**1.88**	**1.10–3.55**	**0.04**
**Surgical margin < 3 mm**		1.27	0.83–1.96	0.26	1.48	0.82–2.67	0.23
**Surgical margin < 5 mm**		1.19	0.60–1.88	0.29	1.41	0.80–2.48	0.23
**Unifocal lesion**		1.21	0.58–2.58	0.60	0.77	0.28–2.14	0.62
**Positive lymph nodes**		1.08	0.71–1.65	0.70	**2.84**	**1.59–5.06**	**0.007**
**Extracapsular spread**		1.28	0.61–2.66	0.50	1.01	0.68–2.07	0.52
**Bilateral groin involvement**		1.27	0.67–2.31	0.48	**2.06**	**1.02–4.16**	**0.04**
**Associated lichen sclerosus**		1.08	0.71–1.65	0.70	1.19	0.68–2.07	0.52
**Associated VIN**	No	1					
	V-HSIL	0.91	0.45–1.82	0.78	0.99	0.46–2.23	0.99
	Differentiated	1.2	0.67–2.13	0.53	0.72	0.21–2.47	0.61
**LVI**		1.09	0.61–1.92	0.77	1.17	0.58–2.34	0.65
**PNI**		1.31	0.85–2.03	0.21	**2.75**	**1.41–5.36**	**0.003**
**Radiotherapy**		0.70	0.52–1.21	0.29	1.28	0.72–2.25	0.39
**Cisplatin**		0.97	0.46–2.06	0.93	0.86	0.55–1.34	0.50

CI: confidence interval; DOI: depth of invasion; FIGO: International Federation of Gynecology and Obstetrics; HR: hazard ratio; PNI: perineural invasion; VIN: vulvar intraepithelial neoplasia; V-HSIL: vulvar high-grade squamous intraepithelial neoplasia.

**Table 4:** Multivariate analysis of factors associated with cancer-specific survival (CSS) (statistically significant factors are highlighted in bold).

Variable		CSS	
	HR	95% CI	*p*-Value
**FIGO Stage III**	1.84	0.90–3.76	0.09
**DOI ≥ 8 mm**	1.25	0.46–3.41	0.66
**Surgical margin (positive)**	**3.13**	**1.37–7.13**	**0.007**
**Positive lymph nodes**	1.28	0.44–3.71	0.97
**Bilateral groin involvement**	1.23	0.43–3.55	0.70
**PNI**	**2.99**	**1.17–7.63**	**0.02**

**Table 5:** Summary of the main studies on the role of PNI in VSCC.

Author	N. of Patients	Setting	Mean Follow-Up	PNI Prevalence	Detection Methods	Survival Outcomes
Rowley [50]	22	Early-stage VSCC (≤2 cm in diameter and <5 mm DOI)	41 months	9.1% (9/22)	H/E	Associated with lymph nodal involvement (*p* < 0.01)
Lerma [55]	71	VSCC (stage I–IV)		21.4% (15/71)	H/E	Shorter survival (*p* < 0.05) at univariate analysis
Holthoff [41]	103 (94 primary VSCC, 9 recurrent VSCC)	Invasive VSCC (stage IB–IV)	28 months (of 49 patients)	52.4% (54/103)	H/E + S100/AE1/3	Independent predictor of recurrence at multivariate analysis (OR: 2.613, *p* = 0.045)
Long [45]	105	Invasive VSCC (stage IB–IV)	45 months	28.6% (30/105)	H/E + S100	Shorter DFS (HR: 2.93, *p* = 0.018) and OS (HR: 3.04, *p* = 0.020) at univariate analysis
Salcedo [48]	421	VSCC (stage I–IV)	67.1 months	7.6% (32/421)	H/E	Independent prognostic factor for OS (HR 2.71; CI: 95% 1.78–4.13; *p* < 0.001) and recurrence-free survival (HR 1.64; CI: 95% 1.08–2.48; *p* = 0.020) at multivariate analysis
Ferrari [46]	74	VSCC (stage I–IV)	45 months	31.1% (23/74)	H/E, S100/AE 1/3 in doubtful cases	Independent prognostic factor for earlier recurrence (HR: 2.74; CI 95% 1.10–7.13; *p* = 0.03) and OS (HR: 4.93; CI 95% 1.33–18.35; *p* = 0.01) at multivariate analysis
Gadducci [49]	64	VSCC (stage I–III)	33 months	25% (16/64)	H/E, S100 in some cases	Prognostic factor for overall recurrence rate (*p* = 0.014), inguinal and/or distant recurrence rate (*p* = 0.001), DFS (*p* = 0.018), and OS (*p* = 0.031) at univariate analysis

CI: confidence interval; DFS: disease-free survival; DOI: depth of invasion; H/E: hematoxylin and eosin; HR: hazard ratio; PNI: perineural invasion OR: odds ratio; OS: overall survival; VSCC: vulvar squamous-cell carcinoma.

## Data Availability

Data are not shared according to privacy/ethical regulations, but are available from the corresponding author upon reasonable request.
